# Evaluation of Delcath Systems’ Generation 2 (GEN 2) Melphalan Hemofiltration System in a Porcine Model of Percutaneous Hepatic Perfusion

**DOI:** 10.1007/s00270-013-0826-5

**Published:** 2014-01-09

**Authors:** Fred M. Moeslein, Elizabeth G. McAndrew, William M. Appling, Nicole E. Hryniewich, Kevin D. Jarvis, Steven M. Markos, Timothy P. Sheets, Rajneesh P. Uzgare, Daniel S. Johnston

**Affiliations:** 1 Department of Diagnostic Radiology and Nuclear Medicine, University of Maryland School of Medicine, 22 South Greene St, Baltimore, MD 21201 USA; 2 Delcath Systems, Inc., 566 Queensbury Ave., Queensbury, NY 12804 USA

**Keywords:** Percutaneous hepatic perfusion, Cancer, Melphalan, Liver, Chemofiltration, Porcine

## Abstract

**Purpose:**

A new melphalan hemoperfusion filter (GEN 2) was evaluated in a simulated-use porcine model of percutaneous hepatic perfusion (PHP). The current study evaluated melphalan filtration efficiency, the transfilter pressure gradient, and the removal of specific blood products.

**Materials and Methods:**

A porcine PHP procedure using the GEN 2 filter was performed under Good Laboratory Practice conditions to model the 60-min clinical PHP procedure.

**Results:**

The mean filter efficiency for removing melphalan in six filters was 99.0 ± 0.4 %. The transfilter pressure gradient across the filter averaged 20.9 mmHg for the 60-min procedure. Many blood components, including albumin and platelets, decreased on average from 3.55 to 2.02 g/dL and from 342 to 177 × 10.e3/μL, respectively, during the procedure.

**Conclusion:**

The increased melphalan extraction efficiency of the new filter is expected to decrease systemic melphalan exposure. In addition, the low transfilter pressure gradient resulted in low resistance to blood flow in the GEN 2 filter, and the changes to blood components are expected to be clinically manageable.

**Electronic supplementary material:**

The online version of this article (doi:10.1007/s00270-013-0826-5) contains supplementary material, which is available to authorized users.

## Introduction

**Fig. 1:**
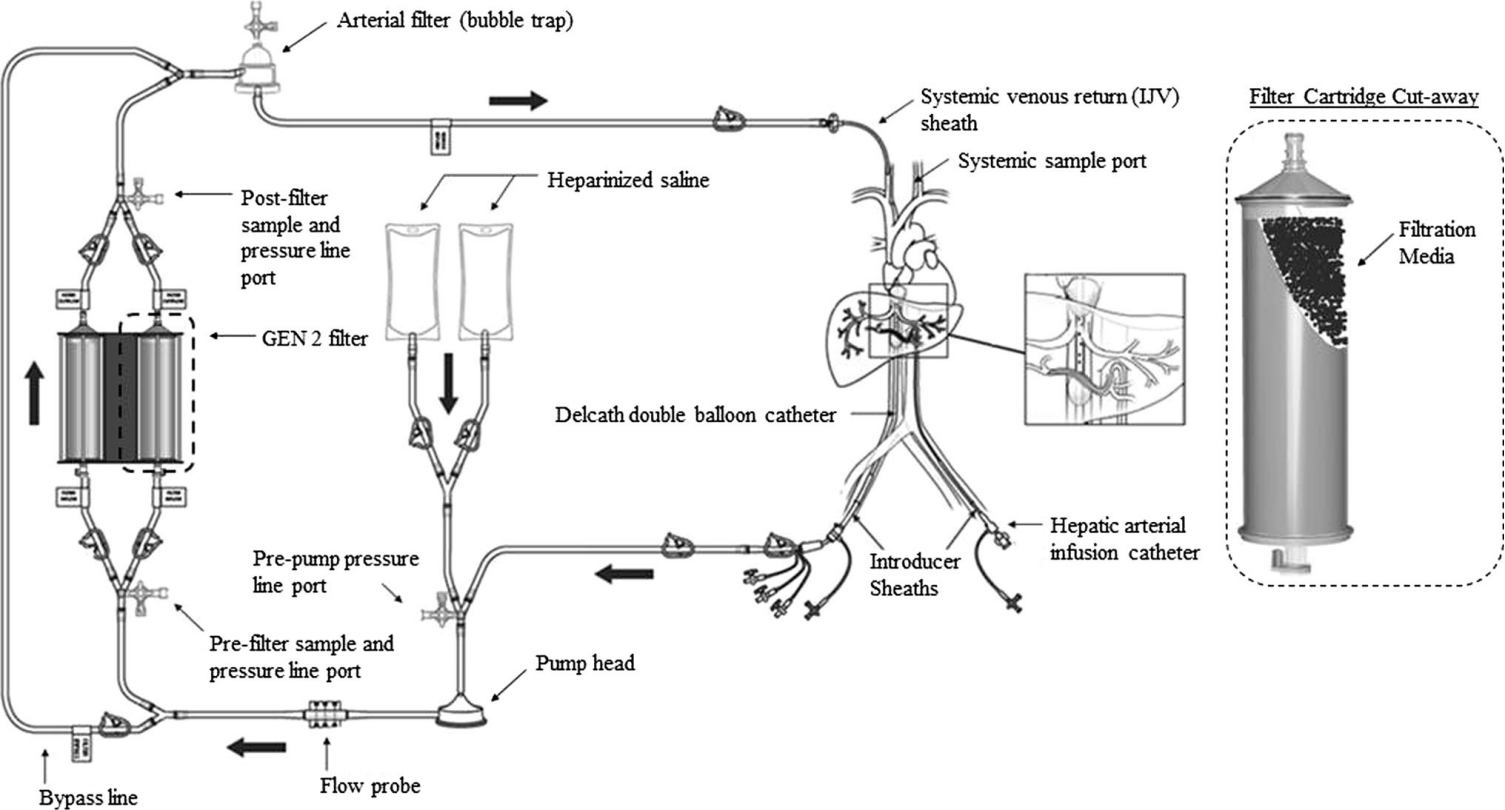
Schematic of the PHP procedure. Chemotherapeutic is delivered through an infusion catheter placed in the hepatic artery. A double-balloon catheter is placed in the IVC to isolate the hepatic veins. Fenestrations between the balloons aspirate venous hepatic blood into the extracorporeal circuit (EC) through Delcath’s hemofiltration cartridges. An arterial filter acts as a bubble trap to ensure that no air embolisms return with the cleaned blood into the jugular vein. A schematic of a filter cartridge is shown on the *right* with a cut-out to show the filtration media within

Regionalized and organ-specific methods for chemotherapeutic treatment limit the toxicity of systemic chemotherapy by facilitating high local drug concentrations at the site of the malignancy compared with systemic administration [1]. The liver has been a primary target for these methods for >50 years based on the high frequency of primary and metastatic liver cancer, the ability to clinically isolate the hepatic vasculature, and the capacity of the liver to withstand toxic insult [2]. Because liver metastases receive nutrients primarily by the hepatic arterial supply as opposed to the portal vein [3], treatment by way of the arterial system has been the primary route of administration for direct high-dose chemotherapy [4, 5]. When regionally administered high-dose chemotherapy is paired with high-efficiency drug filtration of venous outflow, extraregional systemic exposure can be further minimized [4, 6]. This approach forms the basis for percutaneous hepatic perfusion (PHP) (see Fig. 1).

In PHP, filtration efficiency is one of the primary determinants of systemic exposure, and improvements in the filtration system are expected to decrease the amount of drug circulating in the body [4]. To that end, a new filter was recently developed (GEN 2 filter; Delcath Systems, New York, NY, USA), which showed improved filtration efficiency during preclinical in vitro testing. We hypothesized that during in vivo preclinical studies, this filter would have improved filtration efficiency compared with previous filtration systems used during clinical trials [4]. The new filter was evaluated in a porcine model of PHP using melphalan hydrochloride to evaluate filtration efficiency in an in vivo setting before use in the clinical setting. Melphalan filtration efficiency, systemic exposure, transfilter pressure gradient, and the effect of hemofiltration on blood parameters were evaluated.

## Materials and Methods

The protocol for this study was reviewed and approved by an Institutional Animal Care and Use Committee at the testing site where the study was performed. The study was performed in September 2011. A board certified interventional radiologist and perfusionist performed all procedures with appropriately trained scientists and veterinary technicians.

Animals and preoperative care as follows: Six Yorkshire Cross pigs (5 months of age, approximately 78–91 kg) were treated by way of PHP procedure with 220 mg of melphalan hydrochloride. Animals were acclimated to the facility for a minimum of 72 h before the study. Food was withheld approximately 12–24 h before surgery. Water was withheld the morning of surgery. General anesthesia was induced, and a cuffed endotracheal tube was inserted. An intravenous catheter was placed in a vein of the right ear for fluid and heparin administration. General anesthesia was maintained with isoflurane delivered in oxygen, and a ventilator was used to assist respiration.

### Surgical Procedure

Using standard cut-down techniques, an introducer sheath was placed in the femoral vein for insertion of the double-balloon catheter (DBC), into the femoral artery for insertion of the hepatic arterial catheter (HAC) and monitoring of intra-arterial blood pressure, into the jugular vein for blood return, and into the carotid artery for systemic arterial blood sampling. Once the sheaths were placed, ~35,000 U of heparin was administered. Coagulation was assessed by activated clotting time (ACT) approximately every 15 min with a target of 400 s, and additional heparin (for a total of 35,000 U to 75,000 U/animal) was given as needed throughout the procedure to maintain an ACT of >400 s.

Using fluoroscopic guidance, the HAC was placed beyond the gastroduodenal artery. The DBC was positioned in the IVC with the tip at the level of the diaphragmatic hiatus. The DBC was connected to the extracorporeal circuit (EC), which was primed with heparinized saline (2 U/mL), and the venous return sheath was connected to the perfusion adapter. The entire system was purged of air using heparinized saline.

The two balloons of the DBC were inflated with dilute contrast media so the cephalad balloon, which was inflated first, occluded the inferior vena cava (IVC) above the highest hepatic vein and the caudal balloon occluded the IVC below the lowest hepatic vein. Blood flowed through the fenestrations of the DBC into the catheter, to the EC, to the pump through the filter bypass line, and returned to the animal by way of the venous return sheath. The bypass line was then clamped, and blood was directed through the filter. The EC flow rate was adjusted to between 400 and 750 mL/min according to the manufacturer’s instructions for use.

Three minutes after the filter was introduced into the system, 220 mg of melphalan hydrochloride in 500 mL of drug diluent and 0.9 % saline was administered through the HAC during a 30-min “infusion period.” After the infusion of drug, hemofiltration continued for a 30-min “washout period.”

Blood samples were taken to measure melphalan pharmacokinetics (PK). Because the hemofiltration cartridges adsorb nonspecifically, clinical chemistry and hematology samples were taken to evaluate the impact of the procedure on blood chemistry and constituents. Baseline chemistry, coagulation, and hematology sample was obtained immediately after the systemic port was placed and at 6-min intervals throughout the procedure starting from the beginning of hemofiltration. The following blood parameters were analyzed: hematocrit, platelet count, neutrophils, albumin, and fibrinogen. A baseline systemic PK sample was taken immediately before melphalan infusion. Prefilter, postfilter, and systemic samples were taken at 3-min intervals throughout the procedure.

The animals were euthanized under general anesthesia at the end of the procedure. The primary goal of this study was to evaluate filtration efficiency; hence, tissue or organ samples were not collected for clinical pathology. The average time for each treatment was ~2.5 h.

### Sample Evaluation and Analysis

Chemistry, coagulation, and hematology samples were analyzed at Physicians Reference Laboratory (Overland Park, KS, USA). Plasma melphalan concentrations were measured using liquid chromatography–tandem mass spectrometry at Tandem LabCorp (Durham, NC, USA).

The area under the prefilter, postfilter, and systemic concentration–time curves (AUC) from 0 to 60 min was calculated using the linear trapezoidal rule. Overall efficiency was calculated using the AUC_last_ [(pre − post)/pre × 100]. Postfilter samples with melphalan concentration values lower than the level of detection were calculated using linear interpolation. For systemic samples, linear interpolation was deemed inappropriate because some interpolated values were higher than the limit of detection (25 ng/mL). Therefore, values were set to half of the lowest limit of detection (12.5 ng/mL) with the exception of the zero time point, which was assumed to be 0 ng/mL.

### Transfilter Pressure Gradient

Pressure gauges were placed before and after the filter within the EC. Once the filter was online, prefilter and postfilter pressure measurements were taken immediately prior to melphalan infusion and at 3-min intervals throughout the procedure. The transfilter pressure gradient was calculated as the difference between the prefilter and postfilter pressure.

## Results

All six animals successfully underwent the entire 60-min procedure. The mean EC flow rate was 530 ± 34 mL/min.

### Pharmacokinetics

**Fig. 2:**
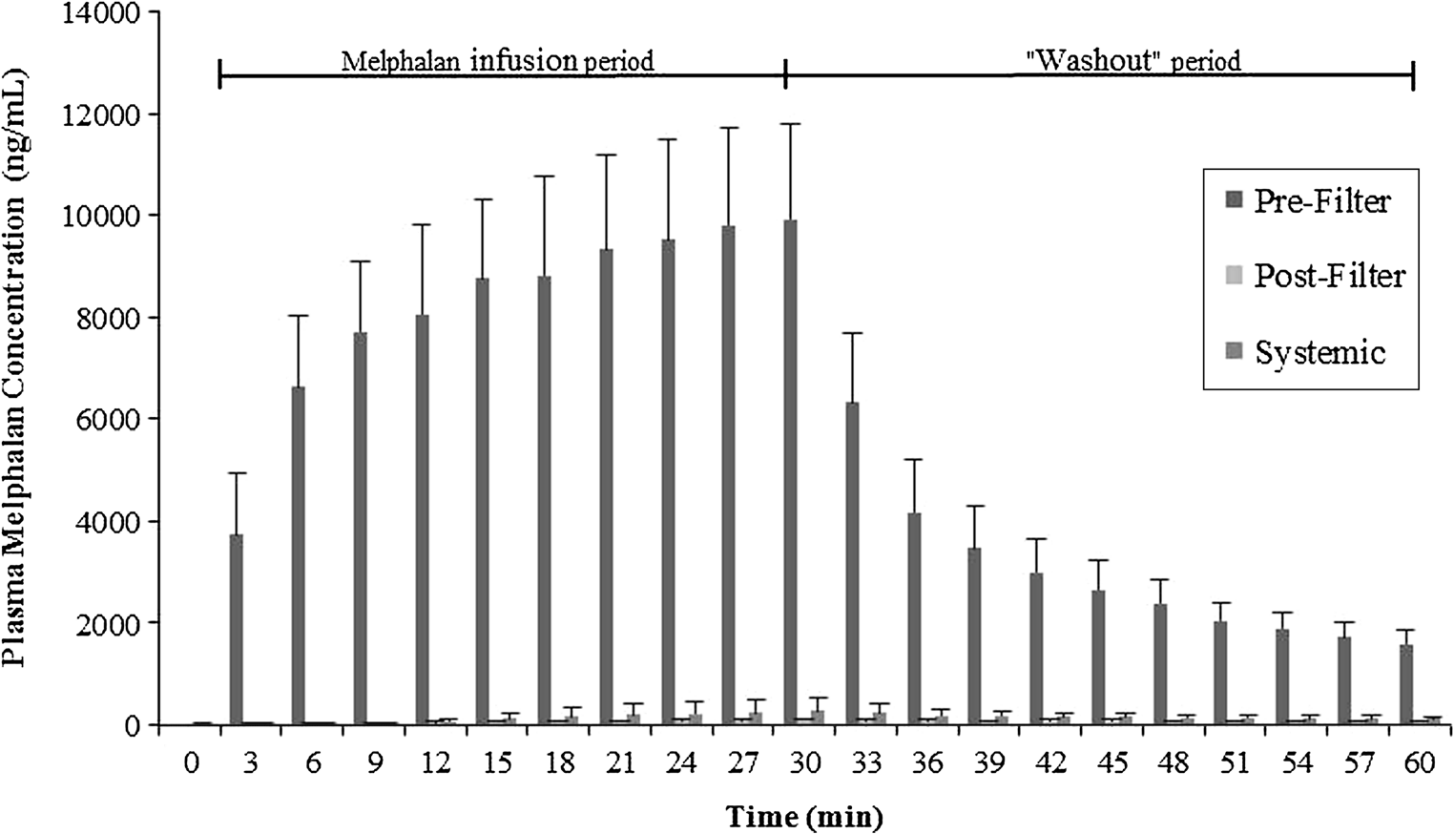
Mean prefilter, postfilter, and systemic plasma melphalan concentrations over time. Samples were taken at 3-min intervals throughout the procedure. *Error bars* represent 1 SD from the mean

**Fig. 3:**
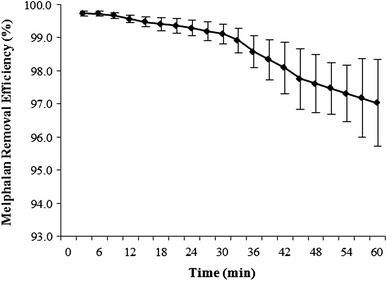
Mean melphalan removal efficiency over time. *Error bars* represent 1 SD from the mean

In general, the prefilter melphalan concentration gradually increased over the course of the infusion period. The mean maximum concentration (*C*_max_) and SD was 10,072 ± 1762 ng/mL, and *C*_max_ typically occurred between 21 and 30 min of infusion. After the infusion period, there was an immediate and continuous decrease in melphalan inlet concentration until the end of the procedure. A summary of the AUC_last_, overall filteration efficiency, and *C*_max_ is shown in Table 1. The mean filter efficiency for the six animals during the 60-min procedure was 99.0 ± 0.4 %. The mean prefilter, postfilter, and systemic plasma melphalan concentrations are displayed in Fig. 2, and the mean plasma melphalan removal efficiency over time is shown in Fig. 3. The full data set of prefilter, postfilter, and systemic plasma melphalan concentration values is available in Supplemental File 1.

**Table 1:** Melphalan pharmacokinetic parameters and extraction efficiency

Parameters and efficiency	AUC (min * ng/mL)			Efficiency (%)	*C*_max_ (ng/mL)		
	Prefilter	Postfilter	Systemic	[(pre − post)/pre × 100]	Prefilter	Postfilter	Systemic
Mean	331,933	3,171	7,921	99.0	10,072	91	265
SD	56,039	873	5,809	0.4	1,762	26	257
Minimum	262,485	2,266	3,871	98.5	8,510	54	101
Median	333,548	3,113	4,721	99.0	9,525	102	128
Maximum	415,215	4,307	17,912	99.5	13,400	119	751

*AUC* area under the concentration–time curve

*n* = 6 animals

### Transfilter Pressure Gradient

The mean pressure gradient across the filter during the entire procedure was 20.9 ± 7.3 mmHg with a prefilter pressure of 50.5 ± 9.4 mmHg and a postfilter pressure of 29.5 ± 10.0 mmHg. The full data set of in-line pressure data are shown in Supplemental File 2.

### Blood Chemistry

The following blood parameters were analyzed: hematocrit, platelet count, neutrophils, albumin, and fibrinogen. The changes in these parameters as a result of the melphalan-PHP procedure with the filter were assessed by comparing values at baseline and at 60 min of filtration (Table 2). The concentration of all parameters decreased between baseline and after 60 min of filtration. The full data set of blood chemistry data are shown in Supplemental File 3.

**Table 2:** Clinical pathology results

Time point	Hematocrit (%)	Platelet count (×10.e^3^/μL)	Neutrophils, Abs (×10.e3/μL)	Albumin (g/dL)	Fibrinogen (mg/dL)
Baseline	30.73 ± 1.62	342.20 ± 60.81	5.07 ± 1.21	3.55 ± 0.23	226.17 ± 32.12
60 min	23.43 ± 3.02	177.00 ± 59.97	1.83 ± 0.25	2.02 ± 0.13	142.83 ± 12.48

All data are mean ± SD

*n* = 6 animals

## Discussion

PHP is a locoregional drug delivery that has been shown in clinical trials to be effective in the treatment of primary and metastatic hepatic neoplasms [4, 7–9]. This technique uses a minimally invasive and repeatable approach to partially isolate the hepatic circulation and deliver high doses of chemotherapy. Notably, PHP offers two extremely important advantages compared with traditional systemic chemotherapy: (1) the ability to deliver a very high dose of cytotoxic drug specifically to a region of interest; and (2) minimization of systemic exposure resulting in fewer side effects than seen with systemic administration of high concentrations of chemotherapeutic agent.

Despite hemofiltration in PHP, during a phase I clinical trial, bone marrow suppression was still the most common dose- or treatment-limiting side effect [4]. In that study, the calculated melphalan filtration efficiency was 78.5 ± 15.2 % in patients receiving a dose of 3 mg/kg ideal body weight (IBW). Therefore, although the filter provided a significant protective effect, a high incidence of melphalan-related side effects was observed. To decrease systemic side effects, a novel filter was designed and showed a melphalan extraction of 98 % in this study.

The GEN 2 hemofiltration system differs from the previous filter generations in that it is comprised of two hemofiltration cartridges in a single housing containing novel, proprietary, free-floating spherical filtration media. The connections to the hemoperfusion circuit have been modified to facilitate the system setup.

The increased melphalan filtration efficiency of the filter resulted in a low systemic exposure throughout the procedure. The mean systemic AUC was 7,921 min * ng/mL. In contrast, systemic AUC in the phase I clinical trial at the 3 mg/kg IBW dose was 37,800 min * ng/mL. A comparison of the phase I study patients treated with 3 mg/kg IBW (dose mean 182 mg; range 153–212 mg, and the animals treated in the current study (220 mg) show that the filter inlet maximal concentrations were similar (*C*_max_ of 11,820–10,072 ng/mL, respectively). The procedure and devices used were similar, with the exception of the filter; therefore the difference in the systemic AUC is attributed to filter’s melphalan filtration efficiency. This increase in filter efficiency of the GEN 2 filter may provide an improved side effect profile compared with previous filters used in PHP trials.

The low systemic exposure seen in these studies raises the question as to whether greater melphalan doses could be used in the system, thus allowing for greater liver/tumor exposure while potentially maintaining or decreasing systemic exposures seen in clinical trials. The phase I study showed that although hepatotoxicity at a dose of 3 mg/kg IBW was manageable/acceptable, an increase to 3.5 mg/kg IBW resulted in a significant increase in grade 3/4 hepatotoxicity [4]; thus, improved efficiency with the GEN 2 filter may not translate to increased maximum tolerated dose.

It is noteworthy that, on average, the systemic melphalan concentration was greater than the melphalan concentration at the filter outlet. The greater ratio of systemic-to-postfilter concentrations with the GEN 2 filter suggest that the amount of drug not captured by the filter system does not represent the majority of drug entering the systemic circulation. One potential source of melphalan in the systemic circulation is leakage by way of perihepatic collateral vessels. Moreover, unlike the human clinical procedure, nonhepatic branches of the celiac artery were not embolized before the procedure because the goals of this acute study were to evaluate the GEN 2 filter with respect to (1) filter efficiency, (2) in-line filter pressures, and (3) extraction of blood parameters, not to perform a complete analysis of systemic exposure. Published reports of clinical studies using hepatic artery infusion and isolated hepatic perfusion procedures, which were the basis for the development of PHP, have reported average leakages of 1.6 and 18 % [10, 11].

Because the study was not a survival study some end points, including bone marrow suppression and clinical histology, were not determined. It remains to be determined whether the expected decrease in systemic exposure occurs clinically and whether it will be sufficient to decrease/eliminate bone marrow suppression. Hepatotoxicity is not expected to be impacted by the GEN 2 filter because the filtration occurs after the liver has been exposed to the injected dose; however, an improved higher efficiency filter may decrease the overall exposure to the liver by decreasing the amount of drug that enters the liver from the general circulation.

Although porcine animal models have been used extensively in preclinical PHP studies, it should be noted that this model differs from the clinical situation in that it is an acute study and uses healthy juvenile animals with undiseased livers. However, it is noteworthy that the prefilter melphalan concentrations observed during the study were consistent with those seen in the phase I study at 3 mg/kg IBW. The amount and concentration of melphalan presented to the filter was comparable with the clinical situation and thus relevant [4]. Measuring the transfilter pressure gradient is a means of monitoring the restriction of blood through the filter. In our experience with two previous filter generations, the transfilter pressure gradients have occasionally exceeded 200 mmHg, the maximum transfilter pressure gradient defined in the IFU for the PHP system. In all development testing and in this study, the new filter design resulted in transfilter pressure gradients <200 mmHg, which is expected to prevent early procedure terminations resulting from low extracorporeal flow and to limit damage to blood cells.

**Fig. 4:**
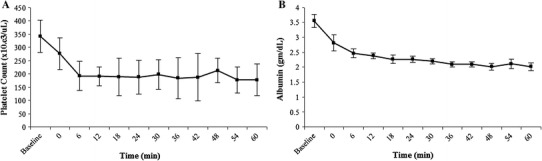
Mean platelet count (**A**) and albumin (**B**) concentrations over time. Error bars represent 1 SD from the mean. © Copyright 2013 Delcath Systems, Inc. All rights reserved

All measured blood parameters decreased during the course of the PHP procedure, especially between baseline and the first 6 min of filtration, and nearly all adsorption occurred within 45 min after filtration started. This sharp decrease in the first 6 min is partly caused by dilution from (1) fluids administered previous and during the procedure to maintain hydrostatic pressure; (2) resident saline used to prime the filter and the EC that entered the animals’ circulation when the filters were brought online; and (3) the 500 mL of saline that was used to deliver the drug. Together, these additional fluids contributed to the decrease in blood component concentrations during infusion, particularly between baseline and 6 min of filtration (Fig. 4). However, it is unlikely that dilution accounts for the total decrease in blood components, and it is expected that filter adsorption of components is a contributing factor. Ku et al. [12] reported similar platelet loss in a doxorubicin PHP trail, and levels returned to normal ranges in 1–2 weeks without intervention. In an isolated hepatic perfusion porcine study with tumor-necrosis factor-α and melphalan reported by Rinkes et al. [13], albumin level decreases were very similar to our study and also returned to normal levels within 1–2 weeks. Although the procedure has a measurable effect on the blood and serum components, it is expected that these changes would be clinically manageable using standard techniques currently used in post-PHP patient management (i.e., administration of fresh frozen plasma, albumin solution, or platelets).

In conclusion, the GEN 2 filter operates within the EC of the PHP system with very low pressure across the filter and allows for enhanced melphalan filtration efficiency in a porcine model. The clinical management required due to the extraction of blood components during the procedure is expected to be similar to that with the previous filtration systems. The improved filtration efficiency of the GEN 2 filter is expected to result in decreased melphalan systemic exposure and corresponding side effects. Future studies may build on this study by performing a full comparison of the GEN 1 and GEN 2 filtration systems and including evaluation of catecholamine extraction to provide insight into expected blood pressure management with the GEN 2 filtration system.

## Electronic supplementary material

Below is the link to the electronic supplementary material.

Supplementary material 1 (DOCX 23 kb)

Supplementary material 2 (DOCX 24 kb)

Supplementary material 3 (DOCX 22 kb)
