# Supplementary material for: Evaluation of Delcath Systems’ Generation 2 (GEN 2) Melphalan Hemofiltration System in a Porcine Model of Percutaneous Hepatic Perfusion
Source: Cardiovasc Intervent Radiol. 2014 Jan 9;37(3):763–9. doi: 10.1007/s00270-013-0826-5 (PMC4024156; doi:10.1007/s00270-013-0826-5)
Supplement: Supplementary file 1 — Supplementary material 1 (DOCX 23 kb) [file 270_2013_826_MOESM1_ESM.docx]

Supplemental File 1: Melphalan Concentration and Efficiency

|  |  | **Pre-Filter** | **Post-Filter** | **Systemic** | **Efficiency** |
| --- | --- | --- | --- | --- | --- |
| **Animal ID** | **Time (min)** | **ng/mL** | **ng/mL** | **ng/mL** | **%** |
| G4831 | 0 | ― | ― | BLQ<(25.00) | ― |
|  | 3 | 2700 | BLQ<(25.00) | BLQ<(25.00) | 99.07 |
|  | 6 | 4490 | BLQ<(25.00) | BLQ<(25.00) | 99.44 |
|  | 9 | 5290 | BLQ<(25.00) | BLQ<(25.00) | 99.53 |
|  | 12 | 5930 | 32.1 | BLQ<(25.00) | 99.46 |
|  | 15 | 6370 | 46.9 | 38.2 | 99.26 |
|  | 18 | 6020 | 56 | 40.4 | 99.07 |
|  | 21 | 7290 | 68.3 | 45.6 | 99.06 |
|  | 24 | 7370 | 74.7 | 55.4 | 98.99 |
|  | 27 | 7730 | 89.5 | 76.3 | 98.84 |
|  | 30 | 8970 | 93.7 | 76.7 | 98.96 |
|  | 33 | 5720 | 104 | 90.2 | 98.18 |
|  | 36 | 3120 | 73.9 | 88.8 | 97.63 |
|  | 39 | 2980 | 87.8 | 105 | 97.05 |
|  | 42 | 2560 | 90.9 | 126 | 96.45 |
|  | 45 | 2400 | 97.3 | 128 | 95.95 |
|  | 48 | 2250 | 77.3 | 90.6 | 96.56 |
|  | 51 | 1940 | 66.5 | 107 | 96.57 |
|  | 54 | 1800 | 69.8 | 101 | 96.12 |
|  | 57 | 1730 | 99.9 | 118 | 94.23 |
|  | 60 | 1670 | 81.3 | 107 | 95.13 |
| G4832 | 0 | ― | ― | BLQ<(25.00) | ― |
|  | 3 | 3950 | BLQ<(25.00) | BLQ<(25.00) | 99.37 |
|  | 6 | 6620 | BLQ<(25.00) | 37.3 | 99.62 |
|  | 9 | 7750 | 31.6 | 39.3 | 99.59 |
|  | 12 | 8390 | 43.4 | 43.8 | 99.48 |
|  | 15 | 9360 | 54 | 54.2 | 99.42 |
|  | 18 | 10300 | 59 | 58.7 | 99.43 |
|  | 21 | 10200 | 56.1 | 65.4 | 99.45 |
|  | 24 | 10400 | 72.6 | 78.1 | 99.3 |
|  | 27 | 10500 | 87.7 | 96.3 | 99.16 |
|  | 30 | 10300 | 96.9 | 118 | 99.06 |
|  | 33 | 7370 | 107 | 123 | 98.55 |
|  | 36 | 5410 | 84.6 | 99.5 | 98.44 |
|  | 39 | 4260 | 65.7 | 97.6 | 98.46 |
|  | 42 | 3740 | 69.3 | 79.3 | 98.15 |
|  | 45 | 3110 | 62.2 | 89.9 | 98 |
|  | 48 | 2870 | 59.5 | 77.5 | 97.93 |
|  | 51 | 2470 | 50 | 85.9 | 97.98 |
|  | 54 | 2150 | 56.2 | 81.6 | 97.39 |
|  | 57 | 1960 | 48.7 | 79.8 | 97.52 |
|  | 60 | 1780 | 50.8 | 78.1 | 97.15 |
| G4833 | 0 | ― | ― | BLQ<(25.00) | ― |
|  | 3 | 4110 | BLQ<(25.00) | BLQ<(25.00) | 99.39 |
|  | 6 | 6460 | BLQ<(25.00) | BLQ<(25.00) | 99.61 |
|  | 9 | 7630 | 30.1 | 36.6 | 99.61 |
|  | 12 | 8250 | 44.7 | 56 | 99.46 |
|  | 15 | 7990 | 57.1 | 66.3 | 99.29 |
|  | 18 | 7660 | 53.5 | 70.3 | 99.3 |
|  | 21 | 7560 | 61.8 | 92.6 | 99.18 |
|  | 24 | 7940 | 65.8 | 102 | 99.17 |
|  | 27 | 8510 | 81 | 115 | 99.05 |
|  | 30 | 7880 | 99.1 | 128 | 98.74 |
|  | 33 | 4330 | 54.5 | 121 | 98.74 |
|  | 36 | 2880 | 45.4 | 123 | 98.42 |
|  | 39 | 2230 | 35.2 | 113 | 98.42 |
|  | 42 | 2000 | 41.2 | 111 | 97.94 |
|  | 45 | 1730 | 44.3 | 116 | 97.44 |
|  | 48 | 1640 | 41 | 104 | 97.5 |
|  | 51 | 1470 | 45.3 | 103 | 96.92 |
|  | 54 | 1450 | 34 | 85.6 | 97.66 |
|  | 57 | 1280 | 33.8 | 83.3 | 97.36 |
|  | 60 | 1200 | 32 | 77.3 | 97.33 |
| G4834 | 0 | ― | ― | BLQ<(25.00) | ― |
|  | 3 | 2930 | BLQ<(25.00) | BLQ<(25.00) | 99.15 |
|  | 6 | 6280 | BLQ<(25.00) | BLQ<(25.00) | 99.6 |
|  | 9 | 7610 | 33.9 | BLQ<(25.00) | 99.55 |
|  | 12 | 5970 | 44.9 | 79.7 | 99.25 |
|  | 15 | 8490 | 51.5 | 177 | 99.39 |
|  | 18 | 8650 | 71.5 | 235 | 99.17 |
|  | 21 | 9590 | 77.6 | 310 | 99.19 |
|  | 24 | 9480 | 104 | 287 | 98.9 |
|  | 27 | 9570 | 109 | 313 | 98.86 |
|  | 30 | 9480 | 119 | 357 | 98.74 |
|  | 33 | 5890 | 110 | 313 | 98.13 |
|  | 36 | 4340 | 85.5 | 256 | 98.03 |
|  | 39 | 3580 | 79.5 | 258 | 97.78 |
|  | 42 | 3210 | 81.7 | 245 | 97.45 |
|  | 45 | 2970 | 84.5 | 252 | 97.15 |
|  | 48 | 2640 | 79.4 | 227 | 96.99 |
|  | 51 | 2350 | 85.4 | 215 | 96.37 |
|  | 54 | 2200 | 83.5 | 201 | 96.2 |
|  | 57 | 2010 | 67 | 190 | 96.67 |
|  | 60 | 1870 | 67.7 | 179 | 96.38 |
| G4835 | 0 | ― | ― | BLQ<(25.00) | ― |
|  | 3 | 2780 | BLQ<(25.00) | BLQ<(25.00) | 99.1 |
|  | 6 | 7190 | BLQ<(25.00) | BLQ<(25.00) | 99.65 |
|  | 9 | 9070 | BLQ<(25.00) | BLQ<(25.00) | 99.72 |
|  | 12 | 10300 | 28.7 | 28.7 | 99.72 |
|  | 15 | 11100 | 34 | 43.2 | 99.69 |
|  | 18 | 11600 | 39.3 | 53.7 | 99.66 |
|  | 21 | 12300 | 45.9 | 66.5 | 99.63 |
|  | 24 | 12900 | 51.1 | 70.8 | 99.6 |
|  | 27 | 13200 | 63.1 | 96.7 | 99.52 |
|  | 30 | 13400 | 64.9 | 95.8 | 99.52 |
|  | 33 | 8280 | 61.1 | 97.1 | 99.26 |
|  | 36 | 5180 | 52.5 | 101 | 98.99 |
|  | 39 | 4470 | 45.7 | 97.2 | 98.98 |
|  | 42 | 3650 | 40.5 | 94.8 | 98.89 |
|  | 45 | 3260 | 41.5 | 91.8 | 98.73 |
|  | 48 | 2810 | 34.9 | 79.6 | 98.76 |
|  | 51 | 2160 | 33.8 | 69.1 | 98.44 |
|  | 54 | 2120 | 32.1 | 70.8 | 98.49 |
|  | 57 | 1830 | 32.2 | 68 | 98.24 |
|  | 60 | 1610 | 27.3 | 55.8 | 98.3 |
| G4836 | 0 | ― | ― | BLQ<(25.00) | ― |
|  | 3 | 5890 | BLQ<(25.00) | BLQ<(25.00) | 99.58 |
|  | 6 | 8740 | BLQ<(25.00) | BLQ<(25.00) | 99.71 |
|  | 9 | 8940 | 27.7 | 34.1 | 99.69 |
|  | 12 | 9440 | 39.1 | 95 | 99.59 |
|  | 15 | 9190 | 39.1 | 282 | 99.57 |
|  | 18 | 8650 | 39.7 | 448 | 99.54 |
|  | 21 | 9050 | 42 | 559 | 99.54 |
|  | 24 | 9060 | 51.4 | 655 | 99.43 |
|  | 27 | 9370 | 53.5 | 703 | 99.43 |
|  | 30 | 9460 | 54.2 | 751 | 99.43 |
|  | 33 | 6340 | 53.4 | 587 | 99.16 |
|  | 36 | 4040 | 44.1 | 386 | 98.91 |
|  | 39 | 3270 | 39.5 | 292 | 98.79 |
|  | 42 | 2780 | 41.5 | 221 | 98.51 |
|  | 45 | 2440 | 43.6 | 218 | 98.21 |
|  | 48 | 2070 | 40.5 | 183 | 98.04 |
|  | 51 | 1790 | 47.6 | 162 | 97.34 |
|  | 54 | 1610 | 29.3 | 157 | 98.18 |
|  | 57 | 1420 | 28.6 | 145 | 97.99 |
|  | 60 | 1280 | 25.5 | 135 | 98.01 |
