# Supplementary material for: Evaluation of Delcath Systems’ Generation 2 (GEN 2) Melphalan Hemofiltration System in a Porcine Model of Percutaneous Hepatic Perfusion
Source: Cardiovasc Intervent Radiol. 2014 Jan 9;37(3):763–9. doi: 10.1007/s00270-013-0826-5 (PMC4024156; doi:10.1007/s00270-013-0826-5)
Supplement: Supplementary file 2 — Supplementary material 2 (DOCX 24 kb) [file 270_2013_826_MOESM2_ESM.docx]

Supplemental File 2: In-line Pressure Data

| **Animal ID** | **Time (min)** | **Pre-pump** | **Pre-Filter** | **Post-filter** | **Trans-filter** | **Post-bubble trap** |
| --- | --- | --- | --- | --- | --- | --- |
| G4831 | 3 | -111 | 70.8 | 49.6 | 21.2 | 52 |
|  | 6 | -110 | 66.6 | 50.8 | 15.8 | 51 |
|  | 9 | -113 | 67.3 | 51.6 | 15.7 | 56 |
|  | 12 | -113 | 65.4 | 52.8 | 12.6 | 50 |
|  | 15 | -117 | 69 | 50.2 | 18.8 | 51 |
|  | 18 | -117 | 70.1 | 45.5 | 24.6 | 56 |
|  | 21 | -118 | 69.2 | 50.3 | 18.9 | 56 |
|  | 24 | -118 | 68.7 | 50.1 | 18.6 | 56 |
|  | 27 | -119 | 67.8 | 49.4 | 18.4 | 55 |
|  | 30 | -119 | 65 | 45 | 20 | 54 |
|  | 33 | -122 | 65 | 45 | 20 | 51 |
|  | 36 | -122 | 65 | 46 | 19 | 52 |
|  | 39 | -122 | 66 | 46 | 20 | 51 |
|  | 42 | -123 | 63 | 45 | 18 | 49 |
|  | 45 | -123 | 63 | 43 | 20 | 49 |
|  | 48 | -125 | 63 | 44 | 19 | 49 |
|  | 51 | -120 | 68 | 48 | 20 | 55 |
|  | 54 | -121 | 66 | 47 | 19 | 54 |
|  | 57 | -123 | 65 | 45 | 20 | 52 |
|  | 60 | -122 | 64 | 44 | 20 | 52 |
| G4832 | 3 | -115 | 45 | 26 | 19 | 37 |
|  | 6 | -114 | 44 | 26 | 18 | 39 |
|  | 9 | -113 | 44 | 26 | 18 | 40 |
|  | 12 | -114 | 45 | 27 | 18 | 37 |
|  | 15 | -115 | 44 | 28 | 16 | 37 |
|  | 18 | -114 | 44 | 29 | 15 | 40 |
|  | 21 | -113 | 44 | 28 | 16 | 40 |
|  | 24 | -114 | 44 | 29 | 15 | 38 |
|  | 27 | -113 | 44 | 29 | 15 | 40 |
|  | 30 | -114 | 44 | 29 | 15 | 38 |
|  | 33 | -113 | 44 | 29 | 15 | 39 |
|  | 36 | -112 | 44 | 29 | 15 | 40 |
|  | 39 | -114 | 44 | 29 | 15 | 40 |
|  | 42 | -114 | 43 | 28 | 15 | 39 |
|  | 45 | -115 | 45 | 29 | 16 | 40 |
|  | 48 | -112 | 44 | 30 | 14 | 42 |
|  | 51 | -114 | 45 | 30 | 15 | 41 |
|  | 54 | -114 | 47 | 33 | 14 | 42 |
|  | 57 | -112 | 48 | 34 | 14 | 44 |
|  | 60 | DNR^a^ | DNR^a^ | DNR^a^ | DNR^a^ | DNR^a^ |
| G4833 | 3 | -130 | 58 | 39 | 19 | 52 |
|  | 6 | -131 | 55 | 37 | 18 | 54 |
|  | 9 | -131 | 55 | 38 | 17 | 51 |
|  | 12 | -131 | 56 | 37 | 19 | 52 |
|  | 15 | -129 | 56 | 38 | 18 | 54 |
|  | 18 | -128 | 57 | 40 | 17 | 56 |
|  | 21 | -128 | 57 | 40 | 17 | 56 |
|  | 24 | -127 | 57 | 40 | 17 | 56 |
|  | 27 | -127 | 57 | 41 | 16 | 56 |
|  | 30 | -126 | 57 | 40 | 17 | 54 |
|  | 33 | -97 | 41 | 24 | 17 | 42 |
|  | 36 | -96 | 40 | 23 | 17 | 40 |
|  | 39 | -96 | 41 | 26 | 15 | 43 |
|  | 42 | -95 | 39 | 26 | 13 | 41 |
|  | 45 | -95 | 40 | 25 | 15 | 43 |
|  | 48 | -94 | 39 | 24 | 15 | 43 |
|  | 51 | -95 | 39 | 25 | 14 | 42 |
|  | 54 | -95 | 40 | 24 | 16 | 42 |
|  | 57 | -94 | 39 | 23 | 16 | 41 |
|  | 60 | -94 | 39 | 23 | 16 | 41 |
| G4834 | 3 | -108 | 51 | 15 | 36 | 42 |
|  | 6 | -107 | 51 | 16 | 35 | 42 |
|  | 9 | -105 | 54 | 19 | 35 | 44 |
|  | 12 | -102 | 50 | 15 | 35 | 41 |
|  | 15 | -100 | 50 | 15 | 35 | 43 |
|  | 18 | -101 | 51 | 16 | 35 | 42 |
|  | 21 | -101 | 50 | 15 | 35 | 43 |
|  | 24 | -100 | 51 | 16 | 35 | 44 |
|  | 27 | -99 | 51 | 16 | 35 | 43 |
|  | 30 | -99 | 51 | 17 | 34 | 43 |
|  | 33 | -98 | 53 | 18 | 35 | 44 |
|  | 36 | -95 | 54 | 20 | 34 | 46 |
|  | 39 | -95 | 54 | 21 | 33 | 47 |
|  | 42 | -95 | 55 | 21 | 34 | 47 |
|  | 45 | -94 | 55 | 22 | 33 | 49 |
|  | 48 | -98 | 54 | 17 | 37 | 44 |
|  | 51 | -98 | 52 | 17 | 35 | 44 |
|  | 54 | -98 | 51 | 18 | 33 | 44 |
|  | 57 | -99 | 52 | 17 | 35 | 45 |
|  | 60 | -99 | 52 | 17 | 35 | 44 |
| G4835 | 3 | -103 | 56 | 32 | 24 | 50 |
|  | 6 | -99 | 53 | 27 | 26 | 49 |
|  | 9 | -99 | 53 | 27 | 26 | 49 |
|  | 12 | -99 | 52 | 27 | 25 | 49 |
|  | 15 | -99 | 52 | 28 | 24 | 49 |
|  | 18 | -99 | 51 | 28 | 23 | 48 |
|  | 21 | -100 | 52 | 26 | 26 | 49 |
|  | 24 | -100 | 51 | 26 | 25 | 49 |
|  | 27 | -101 | 51 | 26 | 25 | 49 |
|  | 30 | -100 | 52 | 25 | 27 | 50 |
|  | 33 | -100 | 52 | 27 | 25 | 49 |
|  | 36 | -99 | 53 | 27 | 26 | 50 |
|  | 39 | -99 | 53 | 27 | 26 | 49 |
|  | 42 | -99 | 53 | 27 | 26 | 49 |
|  | 45 | -99 | 53 | 27 | 26 | 49 |
|  | 48 | -99 | 53 | 26 | 27 | 49 |
|  | 51 | -98 | 54 | 28 | 26 | 50 |
|  | 54 | -98 | 54 | 28 | 26 | 50 |
|  | 57 | -98 | 53 | 29 | 24 | 50 |
|  | 60 | -99 | 53 | 28 | 25 | 50 |
| G4836 | 3 | -97 | 40 | 25 | 15 | 38 |
|  | 6 | -98 | 41 | 28 | 13 | 38 |
|  | 9 | -97 | 42 | 27 | 15 | 39 |
|  | 12 | -96 | 43 | 27 | 16 | 39 |
|  | 15 | -95 | 44 | 27 | 17 | 39 |
|  | 18 | -94 | 42 | 27 | 15 | 40 |
|  | 21 | -94 | 42 | 27 | 15 | 40 |
|  | 24 | -88 | 38 | 24 | 14 | 37 |
|  | 27 | -88 | 38 | 23 | 15 | 38 |
|  | 30 | -86 | 37 | 23 | 14 | 38 |
|  | 33 | -88 | 37 | 23 | 14 | 38 |
|  | 36 | -88 | 37 | 23 | 14 | 38 |
|  | 39 | -88 | 37 | 23 | 14 | 37 |
|  | 42 | -88 | 37 | 23 | 14 | 37 |
|  | 45 | -88 | 37 | 24 | 13 | 37 |
|  | 48 | -89 | 37 | 24 | 13 | 37 |
|  | 51 | -89 | 37 | 23 | 14 | 37 |
|  | 54 | -89 | 37 | 23 | 14 | 37 |
|  | 57 | -89 | 37 | 23 | 14 | 38 |
|  | 60 | -88 | 37 | 23 | 14 | 38 |

^a^Did not record (DNR), circuit was turned off prior to reading being recorded.
