# Supplementary material for: Evaluation of Delcath Systems’ Generation 2 (GEN 2) Melphalan Hemofiltration System in a Porcine Model of Percutaneous Hepatic Perfusion
Source: Cardiovasc Intervent Radiol. 2014 Jan 9;37(3):763–9. doi: 10.1007/s00270-013-0826-5 (PMC4024156; doi:10.1007/s00270-013-0826-5)
Supplement: Supplementary file 3 — Supplementary material 3 (DOCX 22 kb) [file 270_2013_826_MOESM3_ESM.docx]

Supplemental File 3: Select Blood Parameters of Clinical Chemistry and Hematology

|  |  | Hematocrit | Platelet Count | Neutrophils, Abs | Albumin | Fibrinogen |
| --- | --- | --- | --- | --- | --- | --- |
| Animal ID | Time Point (min) | % | x10.e3/uL | x10.e3/uL | gm/dL | mg/dL |
| G4831 | Baseline | 29.3 | 338 | 5.2 | 3.6 | 212 |
|  | 0 | 24.7 | 339 | 2.7 | 2.8 | 159 |
|  | 6 | 22.5 | 260 | 2.8 | 2.4 | 168 |
|  | 12 | 22.7 | 227 | 2.3 | 2.3 | 142 |
|  | 18 | 22.5 | 263 | 1 | 2.3 | 154 |
|  | 24 | 22.3 | 237 | 0.9 | 2.2 | 142 |
|  | 30 | 22.5 | 269 | 1.2 | 2.2 | 140 |
|  | 36 | 22.5 | 264 | 1.3 | 2.1 | 140 |
|  | 42 | 22.9 | 264 | 1.5 | 2.1 | 139 |
|  | 48 | 23.4 | 235 | 2.1 | 2.1 | 137 |
|  | 54 | 24 | 226 | 2.7 | 2.1 | 129 |
|  | 60 | 24.7 | 222 | 1.8 | 2.1 | 140 |
| G4832 | Baseline | 32.2 | 293 | 6.2 | 3.9 | 284 |
|  | 0 | 26.6 | 198 | 4.9 | 2.9 | 202 |
|  | 6 | 24.9 | 151 | 3.7 | 2.7 | 189 |
|  | 12 | 24.1 | 161 | 3.9 | 2.5 | 210 |
|  | 18 | 24.5 | 99 | 3.4 | 2.4 | 177 |
|  | 24 | 26.2 | 101 | 3.3 | 2.4 | 167 |
|  | 30 | 26.1 | 145 | 3.2 | 2.3 | 157 |
|  | 36 | 25.5 | 92 | 2.6 | 2.2 | 160 |
|  | 42 | 25.6 | 69 | 3.3 | 2.2 | 159 |
|  | 48 | 25.6 | 158 | 2.8 | 2.2 | 173 |
|  | 54 | 26.3 | 155 | 2 | 2.3 | 167 |
|  | 60 | 28 | 94 | 2.1 | 2.2 | 155 |
| G4833 | Baseline | 28.5 | DNA^a^ | BLQ^b^ | 3.3 | 236 |
|  | 0 | 19.8 | DNA^a^ | BLQ^b^ | 2.3 | 155 |
|  | 6 | 22.3 | DNA^a^ | BLQ^b^ | 2.3 | 166 |
|  | 12 | 22.5 | DNA^a^ | BLQ^b^ | 2.3 | 175 |
|  | 18 | 20.9 | DNA^a^ | BLQ^b^ | 2.3 | 181 |
|  | 24 | 20.7 | DNA^a^ | BLQ^b^ | 2.1 | 158 |
|  | 30 | 20 | DNA^a^ | BLQ^b^ | 2.1 | 167 |
|  | 36 | 19.5 | DNA^a^ | 1.1 | 2 | 162 |
|  | 42 | 19.1 | DNA^a^ | BLQ^b^ | 2 | 154 |
|  | 48 | 18.5 | 272 | BLQ^b^ | 1.9 | 161 |
|  | 54 | 18.5 | DNA^a^ | BLQ^b^ | 1.9 | 153 |
|  | 60 | 18.7 | DNA^a^ | BLQ^b^ | 1.8 | 156 |
| G4834 | Baseline | 31.7 | 285 | BLQ^b^ | 3.6 | 217 |
|  | 0 | 27.1 | 319 | BLQ^b^ | 2.9 | 166 |
|  | 6 | 24.4 | DNA^a^ | BLQ^b^ | 2.4 | 162 |
|  | 12 | 23.4 | DNA^a^ | BLQ^b^ | 2.3 | 153 |
|  | 18 | 23.2 | DNA^a^ | BLQ^b^ | 2.3 | 147 |
|  | 24 | 22.8 | DNA^a^ | BLQ^b^ | 2.3 | 152 |
|  | 30 | 22 | DNA^a^ | BLQ^b^ | 2.1 | 134 |
|  | 36 | 22 | DNA^a^ | BLQ^b^ | 2 | 130 |
|  | 42 | 21.9 | DNA^a^ | BLQ^b^ | 2 | 128 |
|  | 48 | 22 | DNA^a^ | BLQ^b^ | 1.9 | 124 |
|  | 54 | 22.5 | DNA^a^ | BLQ^b^ | 2 | 130 |
|  | 60 | 22.9 | DNA^a^ | 1.6 | 2 | 129 |
| G4835 | Baseline | 30.3 | 359 | BLQ^b^ | 3.3 | 219 |
|  | 0 | 25.8 | 229 | BLQ^b^ | 2.9 | 184 |
|  | 6 | 22.9 | 145 | BLQ^b^ | 2.4 | 149 |
|  | 12 | 23 | 160 | BLQ^b^ | 2.4 | 161 |
|  | 18 | 23.2 | 173 | BLQ^b^ | 2.3 | 151 |
|  | 24 | 23.2 | 179 | BLQ^b^ | 2.3 | 158 |
|  | 30 | 22.9 | 165 | BLQ^b^ | 2.3 | 144 |
|  | 36 | 23.2 | 151 | BLQ^b^ | 2.2 | 160 |
|  | 42 | 23.5 | 167 | BLQ^b^ | 2.2 | 156 |
|  | 48 | 22.8 | 179 | BLQ^b^ | 2 | 152 |
|  | 54 | 22.9 | 120 | BLQ^b^ | 2.1 | 152 |
|  | 60 | 22.7 | 172 | BLQ^b^ | 2 | 149 |
| G4836 | Baseline | 32.4 | 436 | 3.8 | 3.6 | 189 |
|  | 0 | 28 | 295 | 3.2 | 3.1 | 167 |
|  | 6 | 25.6 | 212 | 2.7 | 2.6 | 151 |
|  | 12 | 25.4 | 215 | 2.7 | 2.5 | 152 |
|  | 18 | 24.5 | 220 | 2.4 | 2 | 137 |
|  | 24 | 24 | 234 | BLQ^b^ | 2.3 | 141 |
|  | 30 | 24.3 | 211 | BLQ^b^ | 2.2 | 127 |
|  | 36 | 23.9 | 229 | 2.3 | 2.1 | 137 |
|  | 42 | 24.3 | 248 | 2.5 | 2.1 | 131 |
|  | 48 | 23.9 | 221 | BLQ^b^ | 2 | 127 |
|  | 54 | 23.8 | 207 | BLQ^b^ | 2.3 | 118 |
|  | 60 | 23.6 | 220 | BLQ^b^ | 2 | 128 |

DNA^a^ – Data not available. Platelet count not available due to clumping.

BLQ^b^ –Below limit of quantitation. No neutrophils counted.
